# Maternal depression and child severe acute malnutrition: a case-control study from Kenya

**DOI:** 10.1186/s12887-018-1261-1

**Published:** 2018-09-03

**Authors:** S. Haithar, M. W. Kuria, A. Sheikh, M. Kumar, A. Vander Stoep

**Affiliations:** 1 Department of Psychiatry, College of Health Sciences, University of Nairobi, P.O. Box 103140, Nairobi, 00101 Kenya; 2 Research Department of Clinical, Health and Educational Psychology, University College London, London, WC1E 7HB UK; 3 Department of Clinical Medicine and Therapeutics, College of Health Sciences, University of Nairobi, P.O. Box 19676, Nairobi, 00202 Kenya; 4 Psychiatry & Behavioral Sciences and Epidemiology, 6200 NE 74th Street, Suite 210, Seattle, WA 88115-1538 USA

**Keywords:** Maternal depression, Child malnutrition, Kenya, Case control study, Poverty

## Abstract

**Background:**

Depression is the leading cause of disease-related disability in women and adversely affects the health and well-being of mothers and their children. Studies have shown maternal depression as a risk factor for poor infant growth. Little is known about the situation in Sub-Saharan Africa. The aim of our study was to examine the association between maternal depression and severe acute malnutrition in Kenyan children aged 6–60 months.

**Methods:**

A matched case-control study was conducted in general paediatric wards at the Kenyatta National Hospital. The cases were children admitted with severe acute malnutrition as determined by WHO criteria. The controls were age and sex-matched children with normal weight admitted in the same wards with acute ailments. Mothers of the cases and controls were assessed for depression using the PHQ-9 questionnaire. Child anthropometric and maternal demographic data were captured. Logistic regression analyses were used to compare the odds of maternal depression in cases and controls, taking into account other factors associated with child malnutrition status.

**Results:**

The prevalence of moderate to severe depression among mothers of malnourished children was high (64.1%) compared to mothers of normal weight children (5.1%). In multivariate analyses, the odds of maternal depression was markedly higher in cases than in controls (adjusted OR = 53.5, 95% CI = 8.5–338.3), as was the odds of having very low income (adjusted OR = 77.6 95% CI = 5.8–1033.2).

**Conclusions:**

Kenyan mothers whose children are hospitalized with malnutrition were shown in this study to carry a significant mental health burden. We strongly recommend formation of self-help groups that offer social support, counseling, strategies to address food insecurity, and economic empowerment skills for mothers of children hospitalized for malnourishment.

## Background

The health and well-being of children is inextricably tied to their early social and emotional experiences. Since feeding and caring for the young is primarily the mother’s responsibility, poor maternal physical or mental health can adversely affect nutrition, health, and psychological well-being of children [1]. The impact of maternal mental health on children’s long-term emotional, cognitive and behavioral problems has been well studied in high income countries [2–4]. However, the impact on child physical health and development has received less attention, especially in low and middle income countries (LMIC) where poor growth due to under-nutrition is a major problem.

Globally, nearly 50.6 million children under the age of five are malnourished; 90% of these children reside in LMIC [5]. Physical growth is a key indicator of child health and nutritional status [6]. Rapid physical growth and development occur in the first two years of life when children are the most dependent on caregivers for meeting their nutritional needs [7]. Studies have shown that healthy maternal behavior and attitude have an essential role in maintaining healthy nutrition in children [5–7].

Depression is the leading cause of disease-related disability among reproductive aged women, globally [8]. The first year after a woman gives birth to an infant is a particularly high risk time for the occurrence of depression. Postpartum depression (PPD) prevalence estimates vary from 15 to 57% [9–13]. An estimated 10–15% of mothers who reside in high income countries are affected [13], with nearly double the prevalence reported in South Asia (Pakistan 28%, India 23%) [9, 11, 14–16]. PPD prevalence estimates from sub-Saharan Africa range from 6 to 30% [17].

Most research on maternal depression and child nutritional outcomes in LMIC has been conducted in S. Asia, where the majority of the world’s underweight children reside [9, 10, 14, 18]. The South Asian studies suggest that poor maternal mental health, particularly maternal depression, is a risk factor for inadequate growth in young children. A case-control study conducted in South India reported a significant association between current major depression in mothers and malnutrition in children (OR = 3.2, 95% CI 1.1–9.5) [16, 19]. A cohort study conducted by Rahman et al. (2007) [20] in rural Pakistan found that perinatal depression in mothers predicted poorer growth and higher risk of diarrhea in infants [21].

In Brazil de Miranda et al. (1996) conducted a case-control study of women of San Paulo and found high levels of psychiatric morbidity among women with protein energy malnourished children, with 63% of cases having high levels of mental distress compared to 38% in controls (OR = 2.8;95% CI =1.2–6.9) [22]. In Rio de Janeiro Hassan et al. (2016) found that maternal mental health was associated with the nutritional status of infants at six months [23]. Infants of depressed mothers were reportedly two standard deviations below average height on WHO norms [12, 24].

A number of studies of child nutritional status in under-fives conducted in sub-Saharan Africa have examined related demographic, socioeconomic and cultural factors [25–29], while a small literature focuses on maternal mental health (Table 1). Adewuya et al. (2008) conducted a case-control study in Nigeria and found that at both three and six months, infants of depressed mothers had statistically significantly poorer growth than infants of non-depressed mothers, with odds ratios of 3.28 and 3.34 for length and 3.21 and 4.21 for height [30]. Depressed mothers reported that they discontinued breastfeeding earlier, and their infants had more episodes of diarrhea and other infectious illnesses [30]. Other sub-Saharan African studies have shown that maternal depression is associated with compromised parenting behavior, non-responsive care- giving practices and decrease in breast feeding, all of which contribute to childhood malnutrition [17, 21, 22]. Ashaba et al. (2015), who conducted a case-control study in Uganda, reported that 42% of mothers of malnourished children were depressed, compared to 12% of mothers of normal weight children admitted to hospital for chronic illness (OR = 2.4; 95% CI =1.18–4.79) [31]. A recent cross-sectional study conducted in Kenya with mothers attending a maternal and child clinic reported a strong association between maternal depression and both non-exclusive breastfeeding (OR = 7.1; 95% CI = 2.9–17.6) and infant underweight status (OR = 4.4; 95%CI = 1.8–11.0) [17]. To date no Kenyan studies have examined the association between severe acute malnutrition (SAM) in infants and depression in mothers. By testing the hypothesis that mothers of children hospitalized with SAM would have a significantly higher likelihood of suffering from depression than children hospitalized with other medical conditions, our research was designed to fill this gap.

**Table 1:** Summary of Key SAM and Maternal Depression Studies in LMIC Contexts

Study	Design	Sample size & methods	Population & setting	Tools & mode of administration	Outcomes (ORs with 95% CI)
Ashaba et al. (2015) [31] Maternal depression and malnutrition in SW Uganda	Matched case control study Not blinded	*N* = 166 children (83 cases and 83 controls); Controls were age and gender-matched chronically ill children.	Rural population from low socioeconomic background. Hospital-based study.	MINI (Mini International Neuropsychiatric Interview) Clinician administered. Children aged 6–60 months.	Prevalence of depression 42% among cases versus 12% among controls OR 2.4 (95% CI = 1.18–4.79; *p* = 0.015)
Ross & Hanlon et al. (2010) [35] Perinatal mental distress & infant morbidity in Ethiopia	Cohort study	*N* = 954 mother child pairs.	Rural population of low socio-economic status. Population-based study.	SRQ 20 (Self- Reporting Questionnaire) Self-administered. Followed up from 3rd trimester through first 2 months postpartum.	Prevalence of High CMD (SRQ20 score > 6) was 9.8% in pregnancy, 2.1% post- natally: persistent high CMD was 2.5% Persistent perinatal CMD was associated with RR 2.15 (95% CI = 1.39–3.24) increased risk of infant diarrhea.
Ejaz et al. (2012) [37] Maternal psychiatric morbidity & childhood malnutrition in Pakistan	Matched case control study Not blinded	*N* = 100 (50 cases, 50 controls with significant co-morbidities were excluded. Controls were children with normal weight. Admitted with common childhood illnesses, like acute respiratory infections, diarrhea.	Urban population in Karachi of low socio-economic status. Hospital based study.	HADS (Hamilton Anxiety and Depression Scale) Clinician administered at time of hospital admission.	Cases were more likely than controls to have depressed mothers OR 0.85 (95% CI = 0.38–1.86; *p* = 0.68)
Rahman et al. (2004) [38] Maternal mental health & childhood growth in Rawalpindi, Pakistan	Case control study Interviewer blinded to case-control status of infant.	*N* = 172(82 cases, 90 controls) Controls were children from same locality whose weight for age was above the 10th percentile.	Urban and peri-urban. Mainly of low SES. Immunization clinic based.	SRQ 20 (Self- Reporting Questionnaire), Self- administered Administered to mother when she came to clinic for child’s 9-mo. immunization	Strong association between maternal depression and poor weight gain. Adjusted OR 2.8 (95% CI 1.2–6.8, *p* < 0.05)
Patel et al. (2003) [14] Maternal depression & infant growth in Goa, India	Cohort study Hospitalized controls.	171 infants age > 9 months 22% with depressed mothers.	Rural population in Goa, India of low SES. Hospital based.	EPDS (Edinburgh Perinatal Depression Scale) Clinician administered at 6–8 week immunization visit.	Babies under the 5th percentile for weight were more likely to have depressed mothers Risk ratio 2.3 (95% CI = 1.1–4.7, *p* = 0.01)
Anoop et al. (2004) [19] Maternal depression as risk factor for malnutrition in children 6–12 months in Kaniyambadi Block, Nadu	Case control study Interviewer blind to child nutritional status.	72 cases and 72 controls, matched. Cases were children 50–80% of expected weight. Controls matched for age, sex, and locality were > 80% of expected weight.	Rural and peri-urban of low SES Community based.	SCID (Structured Clinical Interview for DSM-IV) Clinician administered.	Mothers with malnourished babies were more likely to have post- natal depression OR 7.4 (95% CI = 1.6–3.85; *p* = 0.01)

## Methods

### Study design

We carried out a matched case-control study to examine differences in the prevalence of depression in mothers with young children hospitalized for severe acute malnutrition (SAM) and mothers with young children hospitalized for other health problems.

### Sample

Sample size was determined using open Epi formula for matched case control studies. The minimum number was determined as 74 (37 cases and 37 controls). Estimates from the Husain et al. study (2000) were used to ascertain expected prevalence of maternal depression in children with SAM, and the meta-analytic study by O’Hara et al. (1996) [13] for the expected prevalence of maternal depression in normal children [9, 13].

### Recruitment and consenting procedures

The study was approved by the Kenyatta National Hospital/University of Nairobi ethical review committee (approval no. KNH/ERC/A/180). Consent was administered in English or Kiswahili, depending on the mother’s language preference, and written informed consent was obtained from the participants. Cases were malnourished children ages 6–60 months admitted with severe acute malnutrition at Kenyatta National Hospital pediatric wards between May and June 2014. The controls were gender and age-matched children who were normal weight and admitted to the same hospital for acute ailments. For each case found at the pediatric ward, the first author matched the control on age (up to +/−three months) and sex (except for two pairs). Sampling for the cases and controls was done sequentially in all the pediatric wards at the ratio of one to one. Malnourished children who were admitted for duration longer than 7 days or not within the age range were excluded. Mothers who could not communicate in either English or Kiswahili or were unable to give informed consent were also excluded. Consecutive convenience sampling was applied to obtain cases and controls until the desired sample size was achieved.

### Study procedures

Once consent was given by the mothers, the anthropometric measures of the children were taken using the normogram to ascertain weight and height. Usually the cases had files which categorized these children as SAM. Research data were collected on site in the hospital ward. Two medical students who were trained by first author assisted in the collection of anthropometric data. The mother’s socio-demographic data was captured in the study questionnaire, and the mother was invited for depression assessment. According to study protocol, all mothers whose PHQ-9 scores indicated that they were experiencing severe depression or suicidality were referred to the KNH mental health unit for treatment.

### Data collection instruments

#### Socio-demographic information

We collected information about family income and size, maternal age, education, occupation including spouses’, maternal empowerment and control over finances, HIV status, exposure to chronic illnesses, and family and social support.

#### Measurement of child’s weight and height to confirm case and control status

Once recruited, the children were undressed, and their weights were measured using a digital compression scale and recorded to one decimal point (in kilograms). The height of each child was measured (in centimetres) from crown to heel with the child in prone position using a tape measure. Weight for height scores was generated from WHO normograms, and these were used to confirm the case definition. Children were classified according to WHO criteria (severe wasting (< 70% weight for length or < − 3 Z score) and/or oedema [12]. The cases stood out clearly; therefore there were no changes in classification after anthropometric measurements were taken, and none of the mothers were excluded from the study.

#### Measurement of maternal depression

Maternal depression was assessed using the PHQ-9 which was developed in the U.S. [32]. The PHQ-9 is a self-administered depression scale with nine items that asks about past two weeks with response options ranging from “not at all” to “nearly every day.” The items reflect the nine criteria on which the diagnosis of DSM-V major depressive disorder is based [32]. In this study we used the PHQ-9 to grade depressive symptom severity as none (score of 0–4), mild (5–9), moderate (10–14), or moderately severe/severe (11–27), as recommended by the scale developers [32]. The validity and usefulness of the PHQ-9 in East Africa has been discussed by Gelaye et al. 2014 [33]. The PHQ-9 has been widely used in Kenya, and there is a translated version available in Kiswahili language. Monahan et al. 2009 [34] validated the tool in a Kenyan sample.

The PHQ-9 was administered orally by the lead author or one of her medical school assistants when the mother was unable to complete it (mainly due to poor literacy level) or by herself with the choice of filling the Kiswahili or English version. A high percentage of participants preferred it to be administered orally due to their low literacy level. Quality assurance criteria were instated in training the study assistants in collecting maternal depression information including instructing them to stay close to the tonal/semantic reference of the questionnaire.

### Data analytic approach

The data were entered, cleaned, and analyzed with SPSS version 17. Continuous and categorical variables were analyzed using descriptive statistics. Logistic regression analyses estimated the ratio of the odds of moderate to severe depression (PHQ-9 score ≥ 10) among the cases compared to controls. Adjusted odds ratios were calculated to take into account other risk factors of malnutrition. In analysis of variables with missing data, participants with missing values were excluded.

## Results

We recruited seventy-eight mother-child dyads (39 cases and 39 controls) during the study period. The mean ages of the cases and controls were similar (20.4 months (SD = 12.2) vs 20.3 months (SD = 20.3)), while the mean height (72.8 (SD = 10.4) vs 78.8 (SD = 10.5) and weights (7.2 (SD = 2.2 and 10.1 (SD = 2.8)) of the cases were significantly lower than that of the controls with *t* (76) = − 2.5, *p* < 0.005 (height) and *t* (76) = − 5.2, *p* < 0.001(weight). We recruited 19 girls and 19 boys as cases and 22 boys and 17 girls as controls.

The participating mothers ranged from 16 to 46 years of age, with a mean age of 27.7 (SD = 6.4). With regard to their marital status, 81.2% of the mothers were married; half had had some secondary schooling; and 45.1% were employed. Of the children, 35.1% were firstborn. For those with siblings, the number ranged from 1 to 5. As shown in Table 2, mothers of children hospitalized with malnutrition had significantly lower levels of family income (*X*^2^ = 14.1, df = 2, *p = .001*) than mothers of children hospitalized with other conditions. Among those who were married, mothers with children with SAM were more likely to have spouses who were unemployed. There were no statistically significant differences in mothers of cases and controls with respect to age, marital status, educational attainment, employment status, chronic health conditions, or number of children under age 5 years. Similar proportions of mothers of malnourished children (43.6%) and control children (38.5%) had breastfed their infants for 12 months or more. The self-reported prevalence of HIV was 17.9% among mothers of the cases and 5.1% among mothers of the controls. The majority of the mothers of cases (71.8%) and controls (84.6%) reported that they were receiving social support from family members or friends. Over three quarters of mothers in both groups reported having some control over family finances.

**Table 2:** Baseline Social and Demographic Characteristics of the Mother-child Dyads

Variable	Mothers of Hospitalized Severely Malnourished Children (Cases)	Mothers of Hospitalized Normal Weight Children (Controls)	Difference Statistic	Significance
Mean age (SD)	28.4 (7.6)	27.0 (5.0)	*t (75) = 1.032*	0.32
Mean number of children under age 5 yrs (SD)	1.2 (0.38)	1.3 (0.57)	t (75) = .741	0.46
Marital status N (%)				
Single/divorced/ widowed	9 (23.7)	5 (12.8)	*X* ^*2*^ *(1) = 2.110*	0.28
Married	29 (76.3)	34 (87.2)		
Unknown	1	0		
Mother’s education level				
None	0	1 (2.6)	*X* ^*2*^ *(3) = 3.24*	0.35
Primary	22 (56.4)	15 (38.5)		
Secondary	13 (33.3)	18 (46.2)		
Post-secondary	4 (10.3)	5 (12.8)		
Spouse’s education level*				
Primary	10 (34.5)	11 (32.4)	*X* ^*2*^ *(3) = 1.97*	0.37
Secondary	16 (55.2)	15 (44.1)		
Post-secondary	3 (10.3)	8 (23.5)		
Chronic illnesses (i.e. hypertension, diabetes)				
Yes	5 (12.8)	5 (12.8)	*X* ^*2*^ *(1) = 0.00*	1.00
No	34 (87.2)	34 (87.2)		
Self-reported HIV status				
Positive	7 (17.9)	2 (5.1)	*X* ^*2*^ *(2) = 3.35*	0.20
Negative	31 (79.5)	35 (89.7)		
Unknown	1 (2.6)	2 (5.1)		
Duration of breastfeeding				
≤12 months	22 (56.4)	24 (61.5)	*X* ^*2*^ *(1) = 0.21*	0.64
> 12 months	17 (43.6)	15 (38.5)		
Mother’s occupation				
Unemployed	28 (73.7)	24 (61.5)	*X* ^*2*^ *(1) = 1.29*	0.25
Employed	10 (26.3)	15 (38.5)		
Spouse’s occupation*				
Unemployed	15 (51.7)	8 (23.5)	*X* ^*2*^ *(1) = 5.37*	*0.02*
Employed	14 (48.3)	26 (76.5)		
Family income per annum				
< 36,000	14 (36.8)	1 (2.8)	*X* ^*2*^ *(2) 14.15*	0.001
36,000–150,000	16 (42.1)	18 (52.8)		
> 150,000	8 (21.1)	16 (44.4)		
Social support from others				
Yes	28 (71.8)	33 (84.6)	*X*^*2*^*(1)* = 1.88	0.17
No	11 (28.2)	6 (15.6)		
Mother’s level of control over family finances				
Total control	16 (53.3)	14 (45.2)	*X*^*2*^*(1)* = 0.41	0.52
Partial control	14 (46.7)	17 (54.8)		
None	9 (23.1)	8 (20.5)		

• No data for mothers who are not married (*N* = 14) or marital status is unknown (*N* = 1)

**Fig. 1:**
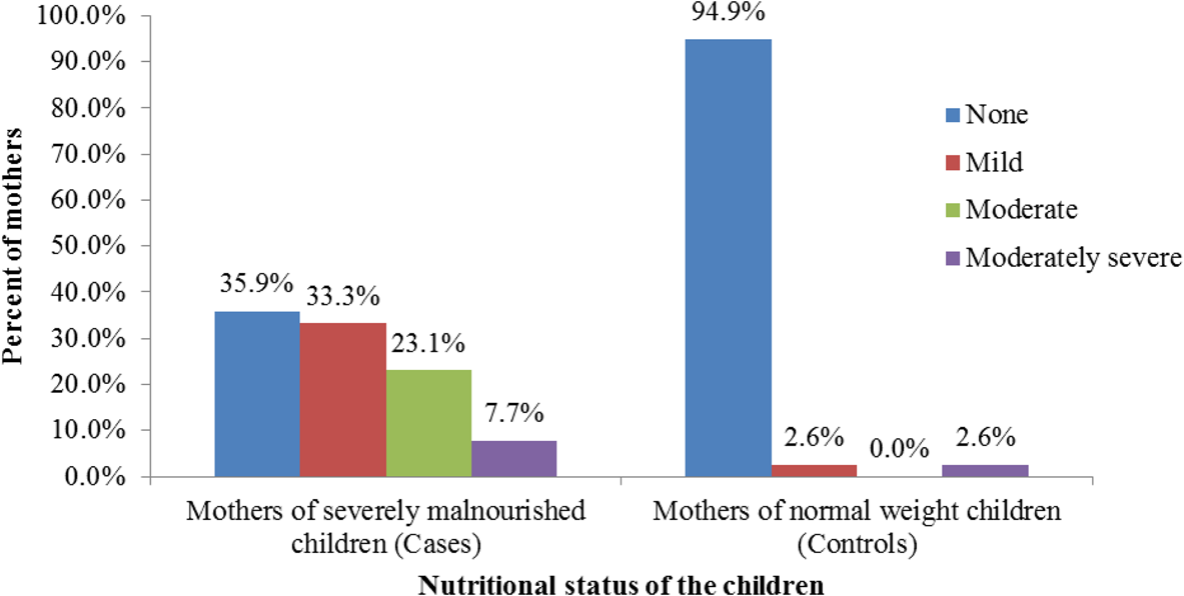
Severity of depression among mothers of cases and controls

The prevalence of mild, moderate, or moderately severe depression was 64.1% (*N* = 25) among mothers of severely malnourished children. This statistically significantly higher than the 5.1% (*N* = 2) prevalence of depression identified in mothers of normal weight children, OR = 33.0; 95% CI 6.9–158.2, *p* < 0.001 (Fig. 1). Among the 25 case mothers who were depressed, 13 had mild depression, 9 had moderate depression and 3 had moderately severe depression. In the control group one mother had mild depression, and the other had moderately severe depression (Fig. 1).

Results of multivariate logistic models showed child nutrition to be significantly associated with maternal depression (AOR = 53.5; 95% CI: 8.5–338.3) and low family income (AOR = 77.6; 95% CI: 5.8–1033.2). Besides family income, none of the covariates were statistically significantly associated with child malnutrition in multivariate analyses (see Tables 2 and 3).

**Table 3:** Logistic Regression Model: Depression Status of Mothers with Children Hospitalized with Severe Acute Malnutrition or Other Health Conditions

Model	Variable	OR (95% CI)	*p* value
1	Depression Status		
	Depressed	33.0 (6.9–158.2)	< 0.001
	Not depressed	1.0	
2	Depression Status		
	Depressed	53.5 (8.5–338.3)	< 0.001
	Not depressed	1.0	
	Family income per annum		
	< 36,000	77.6 (5.8–1033.2)	0.001
	36,000-150,000	3.3 (0.6–18.0)	0.162
	> 150,000	1.0	

## Discussion

Our study demonstrated that infant malnutrition is significantly associated with both maternal depression and family income. Several studies in low income countries such as India, Pakistan, Ethiopia and Uganda have shown similar findings [19, 21, 31, 35]. In a meta-analysis of seventeen studies from eleven different countries, Sukran et al. reported an OR of 2.2 in the association between maternal depression and underweight and an OR of 2.0 in the association between maternal depression and stunting [36]. Our study findings stand out in both the high prevalence of depression in mothers of hospitalized malnourished children and in the discrepancy between the prevalence of depression in these mothers compared to mothers of children hospitalized with other illnesses.

The hospital-based case control study conducted in Pakistan by Ejaz et al. reported high psychiatric morbidity of 50% in the cases, but with nearly as high a prevalence of depression (46%) in controls who were mothers of hospitalized normal weight children [37]. This high mental health morbidity in both cases and controls reflected the generally high prevalence of mental health problems amongst women in Pakistan [38]. Although the prevalence of depression in cases in the current study (64.1%) is higher than estimates of 15–63% reported among mothers in other LMICs, what is more striking in our study is the low prevalence of depression (5.1%) in the controls [14, 16, 21, 30, 31, 39]. A prior Kenyan study conducted by Madeghe et al. (2016) with women with infants attending well-child visits reported a PPD prevalence (EPDS score of 10 or higher) of 13% [17].

Several features of our study sample and methods may have contributed to differences between our findings and those of previous studies of hospitalized children. Our study sample was restricted to mothers whose children had been hospitalized for seven days or less, whereas the children in Ashaba et al. (2015) were not restricted to those with brief length of hospitalization and, subsequently, their control mothers may have been suffering psychological effects of their children’s long hospital stays (as high as 2–3 months) [31]. We only include those children admitted fewer than seven days previously in order to mitigate this potential contributor to maternal distress. The higher prevalence of depression in mothers of our cases may be due to differences in study populations, with the current sample being predominantly urban slum dwellers of low socio economic status, while the Ashaba et al. sample was mainly rural. Table 1 highlights that a variety of tools, including the MINI, EPDS, HADS etc., were used in different studies. We administered the PHQ-9 because it has been validated in Kenya.

Our study had several limitations. Although the study was adequately powered to evaluate the primary study question, the small sample size contributed to the very wide confidence intervals around estimated odds ratios for maternal depression and family income. We were not able to draw conclusions about the contribution to child malnutrition status of factors such as mother’s HIV status and father’s unemployment status that may have distinguished cases from controls in a larger study. Researchers were aware of the case-control status of the mother at the time they administered the depression questionnaire. A high proportion of participating mothers requested that the questionnaire be administered orally. While the medical students were carefully trained to administer the PHQ-9 in a systematic way to both case and control mothers, there may have been errors in understanding the intent of the questions or in the data collectors’ sensitivity, based on the health status of the child or if the mother was perceived as highly distressed. Additionally, because the case-control study was organized around the outcome of the child’s hospitalization, it is difficult to establish temporal sequence between maternal depression and the child’s nutritional status. We were not able to determine which mothers in this study suffered from depression before or during pregnancy. In addition we did not gather information about which children were born preterm or were underweight at the time of their birth. Knowledge of the date of onset and temporal ordering of depression in the mother and malnourishment in the infant would help to determine the optimal timing for targeting intervention strategies.

Having a child who is severely malnourished and who is undergoing hospitalization requires high reserves of parental energy. From what we know about how depression affects functioning, a mother with moderate depression will have difficulty in carrying out ordinary work and social activities. Maternal depression may contribute to undernutrition in children by compromising parenting behavior. Depression can adversely affect the mother’s ability to perform caregiving activities such as breast feeding, stimulation, hygiene and overall care [27]. This interferes with formation of a secure early attachment and bonding behaviors with the baby [19, 21] which, in turn affect a child’s physical and emotional well-being. Conversely, having a child who is severely malnourished is highly distressing. In the current study the malnourished children had been ill intermittently with general deterioration of health that could trigger sustained psychological distress in the mothers. Additionally, the fact that the infant was physically extremely fragile, and this was visually apparent to the mother as she waited for the infant to recover, could heighten feelings of hopelessness and helplessness in the mother. Children in the hospital wards where the study was conducted have high mortality rates with consequences for the mothers’ level of stress and low mood. In contrast, the controls may have been ill for a shorter window of time, so the mothers may not have been subjected to sustained distress.

Our study illustrates the juxtaposition of two health conditions that have serious adverse effects on large segments of populations in low income countries. In this case-control study, we draw attention to tremendous challenges parents face in caring for malnourished children and the burgeoning challenges children face when their caregivers are debilitated with depression. While our study is inconclusive regarding the temporal sequence in the causal association between mother’s depression and child’s nutritional status, the empirical evidence regarding the etiology of depression would support the argument that there is considerable reciprocity, with maternal depression affecting feeding and other child-rearing practices, and the stress of caring for a malnourished child affecting the mental health status of the caregiver [31, 40, 41]. Hospitalization is an added burden on caregiving resources. By matching cases and controls on the condition of hospitalization and by taking family income into account in multivariate analyses, our study was able to control for these sources of parental stress.

Our study findings suggest that mothers of malnourished children are a very vulnerable group for whom emotional health support and economic empowerment programs are warranted. Mothers of malnourished infants are discharged from hospital to carry out feeding protocols that require time, effort, new skills, and financial resources. It may be difficult for mothers with depression and low income to comply with recommendations. Our findings strongly suggest that the need for clinicians who care for families with malnourished infants should learn to recognize and treat maternal mental health conditions that can impede attainment of desired nutritional goals. In addition, the association between malnourishment and very low income calls for measures to ensure that families have adequate economic resources so that mothers and infants do not suffer the health consequences of having insufficient food.

Promising findings have emerged from a trial of the WHO endorsed Thinking Healthy Program conducted in rural Pakistan, where mothers with depression who received cognitive behavioral therapy experienced significant reduction in depression. Additionally their infants had fewer episodes of diarrhea in the first year, compared to women whose depression was not treated [21, 42]. The Thinking Healthy Program targets maternal depression and infant health promotion and has been endorsed by WHO [43] for use in LMIC. The intervention can be offered by lay health workers and could be implemented in Kenya to provide greater support to vulnerable women.

## Conclusions

The prevalence of depression in Kenyan mothers of children under five years of age who were hospitalized for malnutrition was found to be significant. We found maternal depression in these women significantly and markedly higher than in mothers of children hospitalized for other conditions. We strongly recommend formation of hospital-based support and self-help groups for mothers of children hospitalized with severe acute malnutrition. The implementation of WHO endorsed Thinking Healthy Program at community and health care levels to strengthen mothers’ ability to shoulder and share the heavy burden of rearing children who are at risk of life-threatening malnutrition may be considered. In this model, lay health workers including health facility staff can be trained in basic psychosocial support to bolster maternal mental health.

## Abbreviations

DSM: Diagnostic and statistical manual of mental disorders
EPDS: Edinburgh postnatal depression inventory
HADS: Hamilton anxiety and depression scale
LMIC: Lower and middle-income countries
MINI: Mini international neuropsychiatric interview
PHQ-9: Patient health questionnaire 9
PPD: Post partum depression
SAM: Severe acute malnutrition
WHO: World Health Organization
